# The Progressive Approach to EMDR Group Therapy for Complex Trauma and Dissociation: A Case-Control Study

**DOI:** 10.3389/fpsyg.2017.02377

**Published:** 2018-02-13

**Authors:** Ana I. Gonzalez-Vazquez, Lucía Rodriguez-Lago, Maria T. Seoane-Pillado, Isabel Fernández, Francisca García-Guerrero, Miguel A. Santed-Germán

**Affiliations:** ^1^Department of Psychiatry, University Hospital Coruña, A Coruña, Spain; ^2^Assistens Clinic, A Coruña, Spain; ^3^Biomedical Research Institute, A Coruña, Spain; ^4^EMDR Europe Association, Schaffhausen, Switzerland; ^5^EMDR Spanish Association, Madrid, Spain; ^6^Faculty of Psychology, Universidad Nacional de Educación a Distancia, Madrid, Spain

**Keywords:** EMDR, complex trauma, dissociation, group therapy, progressive approach

## Abstract

Eye Movement Desensitization and Reprocessing is a psychotherapeutic approach with recognized efficiency in treating post-traumatic stress disorder (PTSD), which is being used and studied in other psychiatric diagnoses partially based on adverse and traumatic life experiences. Nevertheless, there is not enough empirical evidence at the moment to support its usefulness in a diagnosis other than PTSD. It is commonly accepted that the use of EMDR in severely traumatized patients requires an extended stabilization phase. Some authors have proposed integrating both the theory of structural dissociation of the personality and the adaptive information processing model guiding EMDR therapy. One of these proposals is the Progressive Approach. Some of these EMDR procedures will be evaluated in a group therapy format, integrating them along with emotional regulation, dissociation, and trauma-oriented psychoeducational interventions. Patients presenting a history of severe traumatization, mostly early severe and interpersonal trauma, combined with additional significant traumatizing events in adulthood were included. In order to discriminate the specific effect of EMDR procedures, two types of groups were compared: TAU (treatment as usual: psychoeducational intervention only) vs. TAU+EMDR (the same psychoeducational intervention plus EMDR specific procedures). In pre-post comparison, more variables presented positive changes in the group including EMDR procedures. In the TAU+EMDR group, 4 of the 5 measured variables presented significant and positive changes: general health (GHQ), general satisfaction (Schwartz), subjective well-being, and therapy session usefulness assessment. On the contrary, only 2 of the 5 variables in the TAU group showed statistically significant changes: general health (GHQ), and general satisfaction (Schwartz). Regarding post-test inter-group comparison, improvement in subjective well-being was related to belonging to the group that included EMDR procedures, with differences between TAU and TAU+EMDR groups being statistically significant [χ^2^(1) = 14.226; *p* < 0.0001]. In the TAU+EMDR group there was not one patient who got worse or did not improve; 100% experienced some improvement. In the TAU group, 70.6% referred some improvement, and 29.4% said to have gotten worse or not improved.

## Introduction

Nowadays, EMDR therapy (Shapiro, 1989, 2001) is one of the main treatments of choice for post-traumatic stress disorder (PTSD), as recent meta-analysis have demonstrated (Bisson et al., 2013). Bilateral stimulation (BLS)–characterized by saccadic eye movements, tactile (tapping), or auditory BLS–is a specific component of this type of psychotherapy, and an active contributor to its therapeutic effectiveness (Lee and Cuijpers, 2013).

EMDR is a therapeutic approach structured into eight phases. Phase 1 includes case conceptualization and development of a therapeutic treatment plan. Phase 2 consists of patient stabilization and preparation for further trauma work. Phases 3 to 8 focus on accessing and processing the traumatic memories that are at the core of the presenting problems. Treatment covers past events, present triggers, and future templates.

The use of EMDR in severely traumatized patients with complex trauma and dissociative disorders requires a specific evaluation in Phase 1 and an extended stabilization phase. Different international groups support this phase-oriented model (International Society for the Study of Trauma, and Dissociation [ISSTD], 2011; Cloitre et al., 2012), but a strong debate is taking place in the scientific community regarding the need for specific procedures such as Resource Development and Installation, emotional regulation training, or working with the internal system of dissociative parts (Jongh et al., 2016).

Different authors have proposed adapting the standard EMDR procedure for the treatment of those severely traumatized patients who are included in the complex trauma and dissociation categories (Forgash and Copeley, 2008; Paulsen, 2009; Gonzalez and Mosquera, 2012). A recent review of these adaptations based on the theory of structural dissociation of the personality has been proposed by Van der Hart et al. (2010, 2014a,b). Nevertheless, this area of study lacks systematic research on the use of these EMDR protocols. One of the proposals is the Progressive Approach (Gonzalez and Mosquera, 2012), characterized by gradually approaching traumatic contents. Specifically in Phase 2, psychoeducational work on understanding the general impact of early attachment and trauma, self-care patterns, emotional regulation, and personality fragmentation, is combined with protocols that include BLS. In these protocols, the target to be processed is not a memory; instead, the work focus on dissociative phobias, difficulties in healthy self-care, blockages, and small fragments of traumatic issues. In these interventions, the patient focuses on a self-care image or a dissociative part, noticing the disturbance related to this. BLS is used to desensitize the negative emotions elicited by the target. BLS is also used to reinforce adaptive elements such as resources, adequate self-care, or co-consciousness. In this case, the target is a positive element, and shorter sets of BLS are applied, that usually promotes connection with that resource and reinforces it. The Progressive Approach hypothesis is that this work will promote emotional regulation and dual attention, which are essential for accessing and processing traumatic memories in Phases 3 to 8 of the standard EMDR protocol.

EMDR group therapy is a proposal by Jarero et al. (2006) and Jarero and Artigas (2010). Initially developed for childhood populations, it has also been used successfully with adults, mainly in the context of catastrophes (Jarero and Artigas, 2010; Jarero et al., 2011). In these studies, the patients had been through the same event, thus sharing a common processing target.

In this article, EMDR procedures from the Progressive Approach proposal (Gonzalez and Mosquera, 2012) were tested in a group format on patients with complex trauma and a history of different kinds of intrafamilial childhood trauma and/or gender abuse. Patients had different clinical diagnosis, frequent comorbidity and, many of them, relevant levels of dissociative symptomatology. The main objective was working on stabilization, so treatment was considered as a part of Phase 2. Trauma work was intentionally avoided and would be approached individually. Two types of groups were analyzed, and in one of them specific EMDR protocols were included, such as resource development and installation (RDI; Korn and Leeds, 2002), self-care pattern procedures, and processing of dissociative phobias and blockages (Gonzalez and Mosquera, 2012).

## Materials and Methods

The study was conducted on patients referred to the Trauma and Dissociation Program of A Coruña University Hospital due to an identified history of severe trauma or relevant dissociative symptomatology. The Trauma and Dissociation Program provides a multi-modal approach, including individual therapy (EMDR), family therapy, and trauma-oriented group therapy.

In its initial phase, group therapy focused predominantly on psychoeducation, including information about trauma, attachment, and structural dissociation; emotional regulation; and interpersonal difficulties derived from adverse experiences.

This study attempted to assess whether certain procedures -including BLS- could be introduced in a group setting. Due to the fact that patients in this sample did not share a common event, but did share common difficulties, targets included the latter. Patients in the Trauma and Dissociation Program usually suffer from severe emotional dysregulation and show low functioning levels; thus, procedures were very controlled and directive, but adapted for each patient’s particular characteristics.

The hypotheses to be tested were:

(1) EMDR procedures proposed in the Progressive Approach (Gonzalez and Mosquera, 2012), including BLS, can be used during Phase 2 stabilization in patients with complex trauma and dissociation.
(2) These procedures can be included in a group therapy format.
(3) Specific procedures, such as resource installation, self-care techniques, and processing of dissociative phobias (phobia of dissociative parts, mental contents, change) and blockages can be safe and helpful for this type of patients.
(4) When these procedures are included, the group will experience more benefits than when they are not included.

Bilateral stimulation was performed using tactile stimulation (tapping) instead of eye movements for practical reasons. Self-administered BLS was the predominant modality used due to the difficulty of using eye movements in this setting. The therapists guided the timing, the modality, and the duration of the BLS sets. Patients were provided with minimal information about BLS effects, the therapist explained them some elements of EMDR therapy will be used at some specific moments, and that the effect could be different in different people. This vague description tried intentionally to not suggest any beneficial effect of BLS.

The group was presented as oriented to the consequences of trauma, but not the traumatic memories itself. When these memories emerged, the therapist oriented the patients to the present time and help them to focus on the general topic of the session.

### Sample

Among the different group formats in the Program of Trauma and Dissociation, psychoeducational groups were selected for the study, due to the fact that they share a common structure. This psychoeducational work was considered the TAU condition. All the patients included in the groups were informed about the study, and they consent to participate in it. The content of the sessions was related to the main issues observed in complex traumatization and dissociative disorders (Boon et al., 2011; Gonzalez and Mosquera, 2012; Gonzalez, 2013; Mosquera, 2013). Group work covered the aftermath of trauma related to core beliefs, emotional regulation, and personality fragmentation. Group sessions were structured based on the following topics:

(a) General difficulties to engage in therapy and general rules for the group, emphasizing behavioral activation, and personal commitment to the therapeutic process.
(b) Phobia of future and healthy change, and lack of positive expectations as common consequences of trauma.
(c) Defense mechanisms stuck in trauma time, which become automatisms in the face of non-dangerous triggers.
(d) Understanding personal symptoms and problems, as well as their origins.
(e) Identifying dysfunctional emotional regulation strategies and attachment styles.
(f) Self-care patterns.
(g) Dissociative parts of the personality and core beliefs.
(h) Learning assertiveness and setting boundaries.

EMDR procedures were introduced when therapists considered that the reinforcement of adaptive elements was relevant or when specific dissociative phobias were activated. Working on early traumatic events was intentionally avoided, allowing these memories to be individually processed in EMDR therapy Phases 3–8. Patients had the option of stopping BLS or not using it at any given time. Short sets of tapping were used, and the therapist was in charge of establishing the beginning and the end of each set.

EMDR procedures including BLS were introduced after session 3, gradually increasing the amount of sets per session. The total duration of BLS sets per session did not exceed 10 min. After each set, consisting of 6–8 movements, therapists checked the effect of BLS on every participant, helping with cognitive interweaves as needed.

Patients in both groups (group TAU and group TAU + EMDR) attended additional individual therapy with their psychiatrist and psychologist. Group sessions lasted 90 min, usually on a weekly basis.

### Psychometric Instruments

Instruments covering a wide range of symptomatic areas were used, given that patients presented a variety of clinical diagnoses (depressive, anxiety, bipolar, psychotic, personality, and dissociative disorders) with very different symptomatic profiles. Dissociative symptomatology was specifically evaluated given the recommended precautions when using EMDR with these populations (Fine et al., 1995).

#### Dissociative Experiences Scale (DES)

A 28-item self-administered instrument, developed by Bernstein and Putnam (1986), designed to measure dissociative symptomatology. Items are scored, depending on the frequency of each dissociative experience, in a range from 0 to 100, where 0 represents “never” and 100 “always.” Central points represent 50% of the time. The global score is the sum of the score given to every item, divided by 28. The higher the global score, the more severe the dissociative symptomatology, so improvement is indicated by a decrease in the DES score. The DES has good psychometric properties, with a Cronbach’s α of 0.91 in its Spanish validation (Icarán et al., 1996). Cronbach’s α in our sample was 0.9.

#### General Health Questionnaire (GHQ-28)

Developed by Goldberg and Hillier (1979), this 28-item self-administered questionnaire is designed to evaluate mental health in a broad sense. Answers are to be given in reference to the last few weeks. Items are divided in four sub-scales: A (somatic symptoms), B (anxiety and insomnia), C (social dysfunction), and D (severe depression). Items are scored using values of 0, 0, 1, 1 for the answers. A decrease in the general sub-scales scores represents improvement. In this study, the Spanish version by Muñoz et al. (1979) is used. It was validated by Lobo et al. (1986), showing good psychometric properties, with 84.6% of sensitivity and 90.2% of specificity. Cronbach’s α in our sample was 0.94.

#### Schwartz Outcome Scale (SOS-10)

Developed by Blais et al. (1999), this brief self-report tool measures mental health treatment outcomes (Blais et al., 2011). The Spanish version was developed by Rivas-Vazquez et al. (2001). It has shown to be a reliable measurement of mental health and well-being sensitive to change with treatment. The SOS-10 is a 10-item scale using scores that range from 0 to 6. Improvement is reflected in the increase of the global score. The instrument shows good psychometric properties, with a Cronbach’s α of 0.84–0.96, and good construct validity and applicability in different samples (Young et al., 2003; Haggerty et al., 2010). Cronbach’s α in our sample was 0.89.

#### Analog Scale of Inter-sessions Well-being

Patients evaluate their general well-being in an analog scale, ranging from 0 to 10, in which 0 represents “very bad” and 10 “very good.”

#### Analog Scale of Therapy Session Usefulness

Patients evaluate the general subjective usefulness of therapy sessions in an analog scale ranging from 0 to 10, in which 0 represents “not useful at all” and 10 “very useful.”

### Procedure

Two groups of patients were analyzed (TAU = psychoeducational only, and TAU+EMDR = the same educational work plus EMDR procedures), each one of them composed of several sub-groups. By clinical reasons, each therapeutic group cannot include more than eight patients. BLS was introduced in two of the groups, along with the previously described procedures. In three of the groups, the psychoeducational content was the same, but BLS was not included. These groups were recruited once there were seven patients in the Trauma and Dissociation Program who met the inclusion criteria and accepted to participate in the study. Inclusion was random; it depended only on when each patient arrived to the program and did the initial evaluation. Groups with and without BLS were created alternatively (TAU/TAU+BLS/TAU/TAU+BLS/TAU). Assigning patients to each group was not based on clinical, personal, or sociodemographic characteristics. It was considered that, since the patient’s arrival to the program was entirely random, inter-group homogeneity was guaranteed. Any other kind of randomization would force many patients to have to wait for months to be treated, so it was disregarded for ethical reasons. The Ethics Committee of Galicia approved the study (resolution 2016/279), and all participants signed an informed consent.

The total sample consisted of 31 patients [*M* = 28 (90.3%), *H* = 3 (9.7%)] distributed in a control group (group therapy without EMDR: TAU) and an experimental group (group therapy and EMDR: TAU+ EBL). Group TAU+ EMDR included 14 patients (12 women and 2 men) and group G, 17 patients (16 women and 1 man). Ages ranged from 20 to 59 years.

The inclusion criteria was accepting to participate in a group therapy (some patients with prominent social phobia preferred only individual therapy), having a history of severe traumatization, understanding by this the presence of early severe and interpersonal trauma. Most patients had suffered early intrafamilial abuse (emotional, physical or sexual) and attachment disruptions with their main caregivers. In some cases, there were additional significant traumatizing events in adulthood, such as intimate partner violence, sexual assault, or severe accidents. Early severe traumatization has multiple psychopathological consequences, and clinical diagnoses were diverse. The sample included depressive disorders (*N* = 12), anxiety disorders (*N* = 2), dissociative disorders (*N* = 7), schizoaffective disorder (*N* = 2), substance abuse (*N* = 2), OCD (*N* = 1), conversion disorder (*N* = 2), and PTSD (*N* = 3). Comorbidity was common, and 16 patients met criteria for personality disorder.

Eight patients who met inclusion criteria and participated in some group sessions were not included in the analysis, because they did not attend more than 50% of the sessions. Thus, the amount of treatment was considered insufficient for evaluation. Two other patients did not complete the post-treatment evaluation. From these 10 patients, 6 have been included in the TAU group, and 4 in the TAU+EMDR group.

Mann–Whitney test was used for pre-test and post-test comparison. Wilcoxon signed rank test was used for pre-post intra-group comparisons. Finally, a Chi-square test was performed after recoded variables as improvement/no improvement categories, to analyze post-test results from a clinical perspective.

## Results

### Pre-test Comparison

Patients included in both groups presented a general symptomatology mean of 27.67 (measured with GHQ) and a dissociation mean of 27.64 (measured with DES), indicating significant dissociative symptomatology.

There were no statistically significant differences at pre-test between TAU and TAU+EMDR in dissociative symptoms (DES), general satisfaction (Schwartz), and general well-being using the Mann–Whitney test. Nevertheless, general symptomatology levels -measured using GHQ scores- offered statistically significant differences at pre-test between the TAU and TAU+EMDR groups (*p* = 0.001). The TAU group, as it may be noted, presented more dispersion in GHQ scores, being a less homogeneous group in regards to symptom severity. Statistics are presented in **Table 1**.

**Table 1:** Main pre-test statistics in TAU and TAU+EMDR groups.

	TAU+EMDR				TAU			
	*M*	*SD*	Median	IQR	*M*	*SD*	Median	IQR
General satisfaction (Schwartz)	26.21	9.56	25.0	12.25	21.53	9.76	18.0	14.5
Dissociative symptoms (DES)	25.56	13.37	23.75	22.76	29.35	19.49	25.36	27.85
General health (GHQ)	22.43	4.58	18.5	12.25	32.0	10.23	27.0	18.5

### Pre-post Differences in the TAU+EMDR Group

In the TAU+EMDR group (see **Table 2**), 4 of the 5 measured variables presented significant changes: GHQ general health decreased symptomatology from *M* = 22.428 (*SD* = 4.586) to *M* = 18.642 (*SD* = 6.628); Schwartz general satisfaction increased from *M* = 26.214 (*SD* = 9.56) to *M* = 32.785 (*SD* = 11.053); subjective well-being increased from *M* = 3.357 (*SD* = 2.179) in the first half of the sessions to *M* = 5.578 (*SD* = 2.08) in the second half (effect size: 0.45); and therapy session usefulness assessment changed from *M* = 3.9256 (*SD* = 1.402) in the first half of the sessions to *M* = 5.091 (*SD* = 1.746) in the second half. General health and general satisfaction showed a medium effect size (>5) and subjective well-being and session perceived usefulness a large effect size (>7).

**Table 2:** Wilcoxon signed rank test intragroup pre-post differences.

	TAU+EMDR			TAU		
	*Z*	*p*-Value	Effect size	*Z*	*p*-Value	Effect size
General health (GHQ)	–2.50	0.001	**0.66**	–2.48	0.013	**0.60**
General satisfaction (Schwartz)	–2.48	0.013	**0.66**	–2.29	0.022	**0.55**
Dissociative symptoms (DES)	–0.94	0.345	0.25	–1.28	32.0	0.31
Subjective well-being	–3.30	0.001	**0.88**	–1.28	0.201	0.28
Session usefulness assesment	–2.95	0.003	**0.78**	–1.16	0.246	0.22

*Bold values represent significant values or medium-large effect size.*

### Pre-post Differences in Group TAU

Only 2 of the 5 variables in the G group (see **Table 2**) showed statistically significant changes: GHQ general health (*Z* = -2.479; *p* = 0.013) scores decreased from *M* = 32 (*SD* = 10.228) to *M* = 29 (*SD* = 12.267), and Schwartz general satisfaction (*Z* = -2.294; *p* = 0.022) increased from *M* = 21.529 (*SD* = 9.760) to *M* = 29.058 (*SD* = 13.413). Both variables presented a medium effect size.

### Differences in Compliance with Sessions

The TAU+EMDR group showed less compliance rates. In this group, only 7 out of 14 (50%) attended more than 80% of the sessions. The percentage patients attending more than 80% of the sessions in group TAU was 88.2%: 15 out of 17. These differences are statistically significant [χ^2^(1) = 5.452; *p* = 0.020].

Nevertheless, attending a higher number of sessions does not appear to be related to increase in improvement. Between patients attending more than 80% of the sessions in both groups, 22.7% of them (*N* = 5) stated feeling worse or the same, and 77.3% (*N* = 17) referred feeling better [χ^2^(1) = 14.226; *p* < 0.0001]. All patients attending less than 80% of the sessions (100%, *N* = 9) referred improved well-being.

As discussed below, this result may be related to the lower attendance in the TAU+EMDR group, which on the other hand, presents better results in a higher number of variables. The group using BLS procedures showed less therapeutic compliance (over 50%), but this did not affect clinical improvement. We do not know whether better compliance would have improved results in the TAU+EMDR group.

### Post-test Inter-group Comparison

**FIGURE 1:**
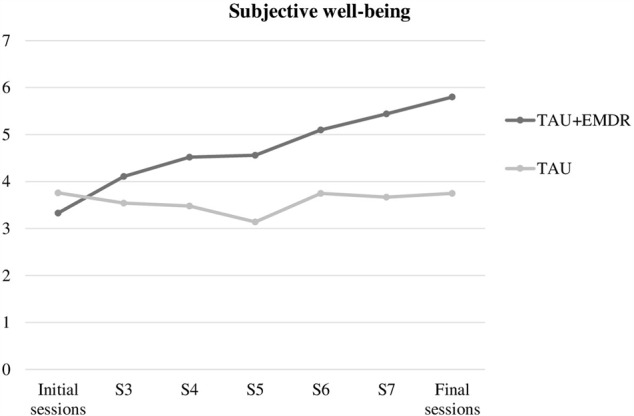
Subjective well-being.

Pre-post comparisons determined the statistical significance reached by inter-group differences. Patients were classified into two categories depending on whether symptoms worsened/not improved or improved. TAU and TAU+EMDR groups were compared. The following results were observed:

Improvement in subjective well-being (**Figure 1**) was related to belonging to the group that included EMDR procedures, with differences between TAU and TAU+EMDR groups being statistically significant [χ^2^(1) = 14.226; *p* < 0.0001]. In the TAU+EMDR group there was not one patient who got worse or did not improve; 100% experienced some improvement. In the TAU group, 70.6% referred some improvement, and 29.4% said to have gotten worse or not improved.

**FIGURE 2:**
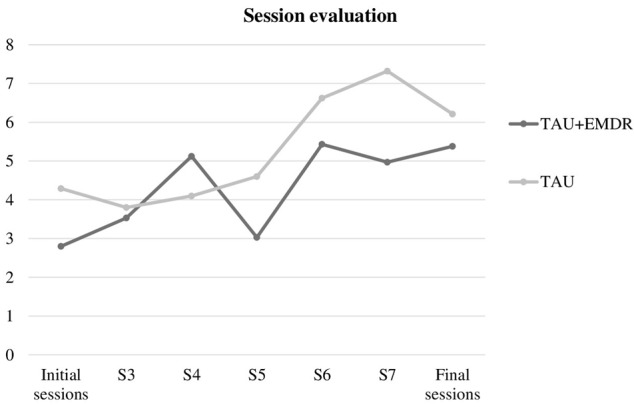
Session evaluation.

In addition, a statistically significant association was found between session subjective usefulness (**Figure 2**) both in the first and second half of the therapy, and belonging either to TAU or TAU+EMDR [χ^2^(1) = 0.9323; *p* = 0.002], with a higher tendency in TAU+EMDR (85.7% vs. 70.6% in TAU) to evaluate sessions in the second part of therapy -which included more EMDR interventions- as more useful. Interestingly, the mean assessment of session usefulness was more irregular in the TAU+EMDR group, with many sessions presenting a lower evaluation, which could be related to the BLS effect of increasing connection with unpleasant emotions.

**FIGURE 3:**
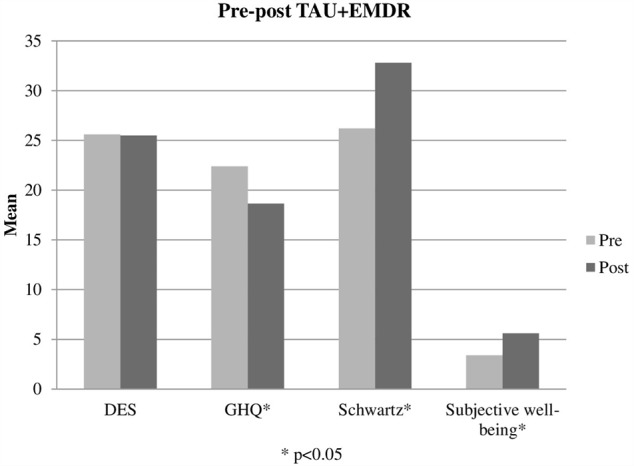
Pre-post TAU+EMDR.

**FIGURE 4:**
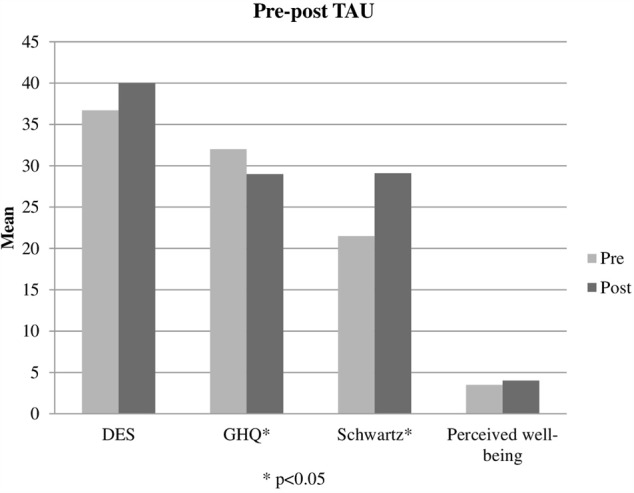
Pre-post TAU.

When comparing other variables presenting pre-post intra-group differences (GHQ and Schwartz) (**Figures 3, 4**), differences between TAU and TAU+EMDR groups did not reach statistical significance. In the Schwartz scale, there was a larger tendency of improvement for TAU+EMDR (71.4% improved their scores) compared to TAU (58.8% improved). Similarly, dissociative symptomatology (using DES scores) decreased 57% in TAU+EMDR and 35.3% in TAU.

An additional post-test inter-group comparison was done analyzing quantitative variables using a Mann–Whitney test. All the variables showed a more positive tendency in the TAU+EMDR, but only general well-being was close to statistical significance (*p* = 0.07) with an effect size (0.32). Effect size for general satisfaction was low (0.19) and also for dissociative symptoms (0.09). General health variable also reached statistical significance (*p* = 0.017) but this variable presented pre-test significant differences. Perceived session usefulness presented an effect size of 0.26.

Interestingly, there was a discrepancy between session usefulness subjective evaluation and changes in well-being. When comparing the first half of the sessions and the second half, the evaluation was higher in the TAU+EMDR group than in the TAU group. But when analyzing each session’s graphics, there is a tendency in TAU+EMDR to evaluate the part of the session including a higher number of BLS procedures as less useful. This tendency changed for the final sessions, in which both groups presented more similarities.

In regards to well-being, the graphic appears completely different. The TAU+EMDR group showed a gradual increase in subjective well-being mean, while the TAU group barely changed throughout the eight therapy sessions.

The analysis of these outcomes supports the clinical impressions from the therapists. Groups including EMDR procedures seemed to evolve more positively, but given that patients suffer from complex trauma and high levels of dissociative symptomatology, BLS sometimes has the effect of increasing the connection with unpleasant emotions and sensations. These patients used to disconnect from those emotions, or showed a tendency to avoid or suppress them.

## Discussion

Results should be analyzed with caution due to the following limitations of the study: groups did not run in parallel, but consecutively, due the characteristics of the Trauma and Dissociation Program. The study was performed in a clinical setting, so it is not a pure research design. Diagnosis was heterogeneous, and a limited number of subjects were included. Contrary to Jarero et al. (2011) proposal, patients did not share an identical traumatic event, but common consequences of different types of severe trauma.

Nevertheless, this study may offer relevant information. Firstly, in a group of severely traumatized people, the application of EMDR procedures that included BLS was safe when used in a very limited and controlled way. The group in which EMDR procedures were applied showed a more positive tendency, with improvement in a higher number of intra-group variables and significant positive differences in inter-group well-being at follow up. General satisfaction showed a positive tendency in this group, though statistical significance was not reached. On the contrary, dissociation remained at similar levels in TAU+EMDR, while increased in TAU, without reaching statistical significance.

Results are modest but relevant, keeping in mind that BLS was used very tentatively, in short sets, only after session 3, and only for a few minutes -a maximum of 10 min-, including preparation for the procedure, patients’ feedback and therapist interventions to contain disturbing material. Eight sessions of group therapy are only a small portion of the therapeutic process required for this kind of patients, so small changes should be valuable.

At the same time -and along with the observations referred by the therapists-, the fact that some of the sessions that included EMDR procedures in the second period were valued as less useful, make us think that patients in this clinical population would show difficulty tolerating longer sets of BLS. Connection with emotions and self-regulation of disturbance is not easy for severely traumatized individuals. EMDR with adapted protocols could be used to promote improvement in this clinical group, but the amount of time allotted for these interventions should be carefully calculated.

Another interesting result was that these specific EMDR procedures, with limited BLS use, were safe for patients with relevant levels of dissociative symptomatology, resulting in a discrete decreasing tendency in DES scores in the group that included EMDR, and some increase in TAU groups.

During group sessions, EMDR therapy was intentionally not described in depth, explaining only that BLS was meant to unblock emotions and sensations. The reason for giving so little information was to avoid the suggestive component in the application of BLS. But at the same time, it could influence the fact that patients in the TAU+EMDR group valued some sessions as less useful. These results favor the need of giving more information in order to prepare the patients for understanding the effects of BLS and manage their emotions and sensations.

Based on the outcomes of this pilot study, a second stage of group therapy will be developed, which will include: specific EMDR preparation, more occasional specific material to promote reflective thinking, and improving patient’s understanding of relevant concepts, such as self-care and personality fragmentation.

## Conclusion

Introducing certain specific EMDR procedures in a group therapy setting for severely traumatized patients appears to be safe and positive. These procedures seem to offer additional benefits to the psychoeducational work oriented toward post-traumatic consequences, when they are included progressively in a very directive and controlled manner. This allows the patient to tolerate connection with disturbing material and assimilate the changes that he or she is experiencing.

## Ethics Statement

This study was carried out in accordance with the recommendations of the Ethics Committee of Galicia with written informed consent from all subjects in accordance with the Declaration of Helsinki. The protocol was approved by the Ethics Committee of Galicia.

## Author Contributions

All authors contributed to the final version of the manuscript. Study design and intervention: AG-V, IF, FG-G, and LR-L; method: MS-P and MS-G; data analysis: AG-V, MS-P and MS-G.

## Conflict of Interest Statement

The authors declare that the research was conducted in the absence of any commercial or financial relationships that could be construed as a potential conflict of interest.
